# A Quantitative Ultrasonic Travel-Time Tomography to Investigate Liquid Elaborations in Industrial Processes

**DOI:** 10.3390/s19235117

**Published:** 2019-11-22

**Authors:** Panagiotis Koulountzios, Tomasz Rymarczyk, Manuchehr Soleimani

**Affiliations:** 1 Engineering Tomography Lab (ETL), University of Bath, Bath BA2 7AY, UK; pik22@bath.ac.uk; 2 Research & Development Centre Netrix S.A., Wojciechowska 31, 20-704 Lublin, Poland; tomasz@rymarczyk.com

**Keywords:** ultrasound computed tomography (USCT), travel-time tomography, sound-speed imaging, liquid compounds, solution concentration, industrial monitoring, industrial reactors, fluid mixing, fermentation, crystallization

## Abstract

This work presents an ultrasound tomography imaging system and method for quantitative mapping of the sound speed in liquid masses. It is highly desirable to be able to inspect vessel fluid mass distribution, notably in the chemical and food industrial operations. Optimization of industrial reactors has been crucial to the improvement of industrial processes. There is a great need to investigate how and if tomographic imaging sensors could aid the automatic control of these process tanks. Single-measurement ultrasound techniques and especially spectrometric methods have been a subject of study of industrial applications. Tomographic systems provide key multi-dimensional and spatial information when compared to the well-established single-channel measurement system. Recently, ultrasound tomography has attracted a great deal of interest in a wide spectrum of industrial applications. The system has been designed as 32 piezoelectric ring-array positioned in a 30 cm tank, with an excitation frequency of 40 kHz. Two-dimensional transmission travel-time tomography was developed to reconstruct the fluid mass distributions. Prior experiments are mainly based on inclusions of a few centimetres and on a liquid solution of different concentrations. They have been conducted to test the spatial and quantitative resolution of the ultrasound imaging device. Analysing the reconstructed images, it is possible to provide accurate spatial resolution with low position errors. The system also demonstrated inclusion movement with a temporal resolution of 4 frames per second (fps) in dynamical imaging sense. Sound velocity quantitative imaging was developed for the investigation of ultrasonic propagation in different liquids. This work, for the first time, shows how quantitative sound velocity imaging using transmission mode time of flight data could be used to characterize liquid density distribution of industrial reactors. The results suggest that ultrasound tomography can be used to quantitatively monitor important process parameters.

## 1. Introduction

Industrial process tomography plays an important role in many sectors of the industry such as manufacturing as well as in food and pharmaceutical operations. The growing necessity to monitor process plant activities leads to the further improvement of online procedures, making tomography an appealing technology. Nowadays, industrial reactors such as chemical reactors, crystallizers, complex stirrers are in a great need of on-line monitoring and automatic control, with the state-of-the-art research positioning on tomographic automated control for optimal solutions.

Ultrasonics have been extensively used for imaging and online monitoring industrial processes. Ultrasounds have a non-destructive and non-invasive property and they can provide quality information as it travels with varying speeds in different media. Devices based on ultrasonic measurements offer many usages in the industrial sector, such as in non-destructive-testing (NDT) [1,2,3]. Ultrasound tomography systems find a great need in conducting pipes and flow/gas imaging, which is a widespread process in the chemical, oil and gas, pharmaceutical and energy industries [4,5,6]. Ultrasonic single-measurement systems have been applied thoroughly in fermentation, solidification, and crystallization processes in the food or pharmaceutical industry [7,8,9]. These techniques have been already proposed as a promising method to determine the density of slurry mixtures, which is important in characterizing the extent of crystallization [10,11,12]. Ultrasonic spectroscopy usually offers a low-cost solution and thereafter is a highly distributed technique in slurry (binary) mixture characterization processes [13,14,15]. Spectroscopy is used, especially, in processes with some phase changes in material, such as the nucleation or solidification in the crystallization process, as ultrasound propagation is being affected by the density and compressibility of the materials. The use of ultrasonics is therefore appropriate to monitor the fermentation process [16]. Moreover, several equations have been proposed concerning ultrasonic velocity in relation to density and compressibility of binary mixtures. Among the most commonly used are the equations of Urick et al. [17].

Nevertheless, tomographic ultrasound imaging offers a great alternative to traditional ultrasonic single measurement techniques, as it provides methods of sampling a substantial volume rather than a single point, with an automated way of data collection and processing. Semi-batchwise tank precipitators are often used in crystallization, fermentation, and other processes of chemical or food industry. Crystals or food solid parts are being produced by mixing miscible liquids. The quality of solid products is based on particle size, morphology, and purity. Fluid interaction is critical to result in good yield. Therefore, suspension uniformity has been always a crucial factor for crystallization and thereafter, the controlling of the stirring and injection process are important parameters. Tomographic solutions tend to be a reliable method of inspecting the distributions of suspension during the injection and mixing process. Earlier works explored the benefits of electrical resistance tomography (ERT) in such setting [18,19,20], which presented good performance. Ultrasound computed tomography (USCT) could be extremely useful to characterize the density of different solutions during prior stages of the process (before nucleation), but can also aid the latter stages by monitoring the distribution of crystalline suspensions. Therefore, USCT is currently under great attention by the research community, especially in crystallization and fermentation processes that occur in batch reactors, applying all new computational aspects of tomography in these specific applications [21,22].

**Figure 1:**
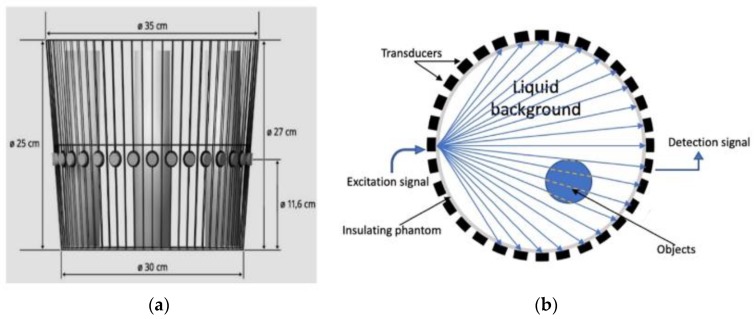
Setup of a ring of 32 ultrasonic transducers in a fan beam architecture. (**a**) One actuated transmitter. (**b**) Measurement’s principle of a 32-electrode USCT system.

This article presents an online monitoring, sound-speed transmission USCT system, for industrial tanks, based on the arrival time of flight (TOF) data. The developed USCT system is divided into three basic components: A multi-piezoelectric sensor, a sensing electronic setup for data acquisition, and a computer system for image reconstruction. Circular pipeline-based sensors measure an entire cross-sectional volume. Figure 1b displays the circle-wise sensor setup and subsequently the “sensing zone” of the tank. The main purpose of this work is to investigate ultrasonic wave propagation in liquids inside industrial reactors. The innovative approach of the sound-speed imaging has the potential to be of great advantage to processes that want to distinguish between liquids of different densities or even liquid/solid particle formations within an industrial tank. Such slurries can easily be found in fermentation and crystallization processes. 

## 2. Transmission Forward Model

This work is based on the transmission mode of actuated pulses. Recording waveforms from the sensors help reconstruct sound-velocity profiles, using the TOF of the first arrival pulse. Figure 1a depicts the experimental tank with the tomographic setup. In Figure 1b, there is a 2D scheme describing the main functionality of transmission tomography. Black squares, positioned in a circular formation, present the transducers. They work as both transmitter and receiver, mounted at the outer boundary of the phantom. The tomographic instrument measures the time needed for a wave to overcome the medium. Figure 1b presents the modelled wavefront’s propagation with the blue lines. Each spherical wave can be approximated by a cone of rays and subsequently, every plane wave by a fan of rays. Using the recorded TOF values, one can calculate the average sound-speed for the path. In some cases, the acoustic properties of materials differ too much, the sound cannot penetrate the inclusion, so the particular pulse is blocked. To be able to model this measurement in tomographic setting, models of ray-based approached are investigated and compared here.

First, a thin line approach leading to “sparse” interaction between image pixels and intersection line is considered. Using the time data and knowing the exact distance, one can compute sound-speed data, which represents the average sound velocity of each one of the waves-rays (1):

(1) $$S=\frac{D}{\Delta m}$$

where D is the distance of the travel-path of each ray, S is the computed average sound velocity of a ray and Δm is the TOF data. The average sound speed of an acoustic wave that travels through path l, between an emitter and a receiver is represented by $s_{m}$ (1 < m < M) and it can be expressed by the following path of a line, where M is the total number of paths in multi-sensor array measurement. This line is expressed by the integral of the spatial distribution of the sound speed of the domain (sound velocity distribution):

(2) $$s_{m}=\int_{l}V\left(l\right)\;dl$$

To algebraically reconstruct data, first of all, one needs to discretize the domain. The equation below describes the reconstruction problem with linear algebra:

(3) $$V_{m}=\sum_{n=1}^{N}w_{m,n}\;s_{n}$$

where $s_{n}$ is the sound speed produced by the $n_{th}$_cell, 1 < n < N and $w_{m,n}$ is the weighted value that describes how much each specific wave-ray affects the domain, namely the pixels. These weighting values have been computed by an algorithm, which checks the distance of the ray to the centre of the pixel. Changing the assigning values process, by treating the pixel as a circle, one can reduce the computation costs [23].

A tomographic inspection consists of many of these rays, whose amount depends on the angle of emission beam. All these ray equations form a system of linear equations, the so-called forward problem.

**Figure 2:**
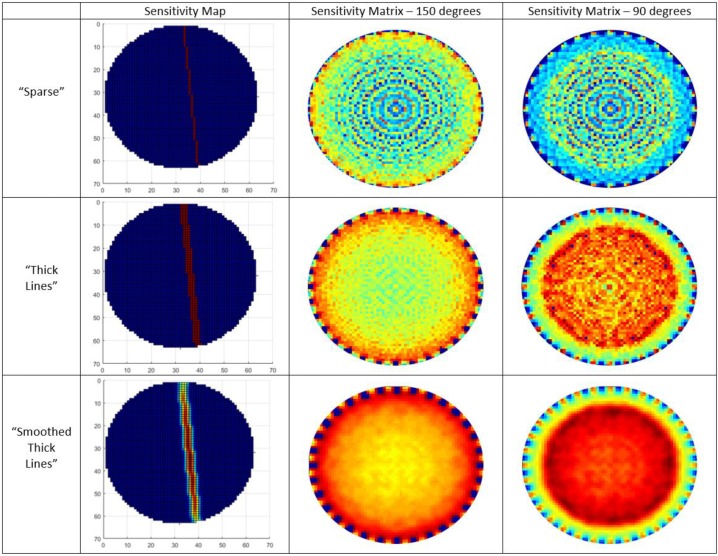
**Left**: Sensitivity maps depicting the different form of ray in each method. **Middle**: Full frames of sensitivity matrices produced by all the proposed methods for 150 degrees of angle of the beam. **Right**: Full frames of sensitivity matrices produced by all the proposed methods for 90 degrees of angle of the beam.

(4) s=A V

where $s={\left[s_{1},s_{2},\ldots ,s_{M}\right]}^{T}$ is the vector of calculated sound-speed values from the measured TOF values, $V={\left[V_{1},V_{2},\ldots ,V_{M}\right]}^{T}$ is the vector that contains the discretized values of the sound-speed spatial-distribution, and A is a M x N  sensitivity matrix, containing the weighted values $w_{m,n}$, and normalised in the range of (0–1) by the sum value in every pixel of the domain. A single frame of matrix A is depicted in Figure 2 in “Sparse” section.

The previous method faces a few important limitations and drawbacks. This straight-ray model has been demonstrated to be inappropriate for obtaining accurate USCT images [24], lacking a good uniform distribution of values, an innate problem of tomographic coverage. The main problem is that this method introduces sparsity in the sensitivity matrix. Another problem is that while the resolution of reconstruction increases, more “ray artefacts” show up in the results. However, regularization techniques could sometimes overcome these artefacts, but they cannot always do so [23]. Therefore, an alternate approach of ultrasound transmission tomography sensitivity kernels was applied. This is the “Thick Lines” method [25], whose single-frame plot is depicted in Figure 2. Regarding this model, the sensitivity map for any two transducers is a straight line with a certain width, equal to the width of the transducer. From experience, the number of transducers, as well as the size of the pipeline are determining the width of the ray [26].

Upon evaluation of the “Thick Lines” full-frame plots, as depicted in Figure 2, many discontinuities could be noticed. Due to this lack of uniformity, another model needs to be considered. The proposed model is based on the “Thick Lines” approach. We call this model “Smoothed Thick Lines” and it has been constructed by smoothing every single map of sensitivity matrix using a Gaussian filter with standard deviation. The smoothing had to account for a specific direction in order to produce a thick line with higher sensitivity in the central region, and gradually decreased sensitivity layers on its left and right. This approach is presented at the bottom of Figure 2. This new sensitivity matrix calculation method provides smoother results, overcoming previously seen discontinuities, as depicted in Figure 2. In this way, its sensitivity full frame is also smoother than in the previous method. This smoothing introduces uniformity in values of distribution, and subsequently, slightly better results even when using single-step reconstruction algorithms. Producing a reliable sensitivity matrix, the angle beam of emission plays a significant role. Having to decide between a case of a full angle of 150 degrees and a half angle of 90 degrees, their full-frame plots have been presented in Figure 2. From these plots one can easily notice that the 90 degrees angle of the beam provides higher sensitivity in the centre and lower in the edges, which is a fact that describes the physical operation of the system. Eventually, the more uniform the sensitivity distribution is in the region of interest (ROI), the higher the spatial resolution gets [27,28].

## 3. Sensitivity Matrix Analysis

**Figure 3:**
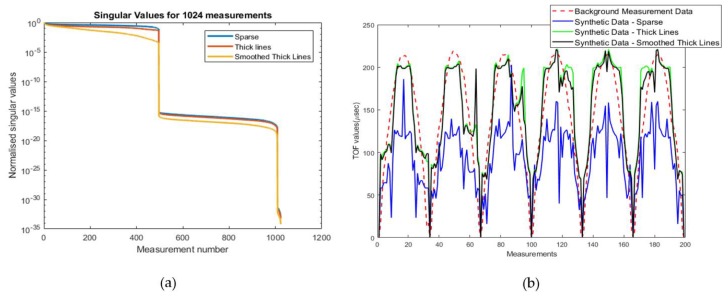
(**a**) Singular Values of all the different methods of the sensitivity matrices. It helps to characterize inverse problems according to their ill-posedness. (**b**) Plots of synthetics data produced by all the different methods of sensitivity matrices against the background measurement.

To evaluate the performance of each of these approaches to the forward and inverse problem in UCSST, an evaluation of the forward model and ill-posedness of the inverse problem is considered. Singular values decomposition (SVD) provide the means to study the ill-posedness of an inverse problem, by decomposing the sensitivity matrix A. SVD of A is

(5) $$A=U\Sigma V^{T}=\sum_{i=1}^{n}u_{i}\sigma_{\iota }v_{i}^{T}$$

where $U=\left(u_{1},u_{2},\ldots ,u_{n}\right)\in R^{mxn}$ and $V=\left(v_{1},v_{2},\ldots ,v_{n}\right)\in R^{mxn}$ are matrices with orthonormal columns called singular vectors, $U^{T}U=V^{T}V=I_{n}$ and the diagonals of Σ includes the singular values, which are positive numbers $\left(\sigma_{1},\sigma_{2},\ldots ,\sigma_{n}\right)$ sorting in non-decreasing order. The plot of singular values decomposition (SVD) of different sensitivity matrices helps to understand the level of ill-posedness. Plotting the base 10 logarithmic scales of the singular values as shown in Figure 3a. Figure 3a displays SVD values of different sensitivity matrices. It is clear that the “Smoothed Thick Lines” approach is more ill-posed when compared to the other two methods. According to this curve, the “Sparse” method is giving us the least ill-posed solution, and on the other hand, the “Smoothed Thick Lines” is giving us the most ill-posed problem. In terms of reconstruction, the best result can be achieved with the “Smoothed Thick lines” method, due to its inherent regularization. The “Sparse” and “Smoothed Thick Lines” techniques are assumed as hard-field and soft-field methods. The “Thick Lines” technique goes in between.

TOF raw measurements could not be well described by a hard-field tomography model as the “Sparse” method, due to wave characteristics and nature. Other reasons for the low spatial resolution could be caused by dimensions of sensors (not being a point source for example), total count, and technical issues. In this case, a system matrix with a slight soft-field approach matches our need. As both “Thick Lines” and “Smoothed Thick Lines” overcome the “Sparse” method. In conclusion, the smoothing factor for computing the sensitivity matrix seems to affect positively the results, making regularization sometimes inessential. 

To evaluate sensitivity matrices further, the values of the experimental background measurement data (without any inclusions to the medium) is compared with the, produced, synthetic background data, using all the different sensitivity matrices. Figure 3b presents experimental data with the blue coloured curve and all synthetic data, from different sensitivity matrices, in other colours. These values are TOF data and always have a repeatable pattern, due to the circular shape of the tomographic setup. Synthetic data have been produced by solving the forward problem, using a uniform sound speed distribution in the ROI. The closer these data get to the experimental data; the more efficient our sensitivity matrix is. Upon observation of signal-forms of four different sensitivity matrices, it is noticeable that “Thick-lines” approach produces better data than the “Sparse” one, and consequently “Smoothed Thick Lines” seems to produce the closest data to the real experimental values.

## 4. Inverse Problem

To reconstruct the image, each sensitivity matrix is multiplied by its corresponding sensor loss value; Similar to sensor loss values back projection to the image plane individually. Then, these matrices are summed to provide the back-projected acoustic velocity distributions, which will be represented by the colour level [29]. The linear inverse problem in travel-time ultrasound tomography can be defined as the retrieval of the change in time of travelling of the pulse Δm from a change in the sound velocity in the domain ΔV. Equation (1) is a linear equation that describes the forward problem principal concept. System matrix A is computed by the foundations of propagation of rays in space, as described in the previous section.

(6) Δm=A ΔV+e

where A is the forward operator and e is the noise in the measurement. ΔV is defined as the acoustic velocity profile of the scanned region in different discrete times and Δm as the TOF measurements. If the inverse of A existed, then Equation (1) could be solved directly. However, traditionally, acoustic tomography presents a much smaller number of measurements than the number of reconstructed image pixels. Subsequently, the sensitivity matrix A is not a square matrix and not invertible. A common technique to solve the inverse problem, is to use the transpose matrix of A.

(7) $$\Delta V\approx A^{T}\;\Delta m$$

Total variation regularization (TV) was introduced by Rudin, Osher, and Fatemi [30]. It is an iterative regularization algorithm. TV de-noising is an effective filtering method for recovering and reconstructing piecewise-constant signals, all while being a deterministic technique, which safeguards discontinuities in image processing tasks [31,32]. The essence of the regularization methods is to transform the solution of Equation (6) into an optimization problem. The total variation problem is defined as:

(8) $$minA\left(\Delta V\right)={\left\|\left(A\;\Delta V+e\right)-\Delta m\right\|}^{2}+a\;{\left\|\nabla \Delta V\right\|}_{1}$$

where a is the regularization parameter, ∇ is the gradient, and ${\left|\left|.\right|\right|}_{1}$ is the $l_{1}$−norm.

TV regularization can be isotropic or anisotropic on the smoothness terms. The isotropic TV norm is L2-based and non-differentiable, while the anisotropic norm is L1-based and less time-consuming. Isotropic regularization schemes relax smoothness constraints at boundaries. Anisotropic formulations let smoothing occur along the borders, but not in a transversal manner [33]. A characteristic difference between these two widely used TV regularization methods is that the isotropic TV is rotationally invariant. The rotational invariance of this method is known to cause geometric distortions, by favouring edge orientations aligned with co-ordinate axes. Isotropic TV regularization is expressed by (9):

(9) $$R_{ITV}\left(V\right)=\sum_{j}{\left\|D_{j}\;V\right\|}_{2}$$

And anisotropic TV regularization is expressed by (10):

(10) $$R_{ITV}\left(V\right)=\sum_{j}{\left\|D_{j}\;V\right\|}_{1}$$

$D_{j}$ represents a finite-difference approximation of the spatial image gradient and V is the image. An isotropic version of the TV functional is given by Equation (11), as used in this work.

(11) $${\left\|\nabla \Delta V\right\|}_{2}=\sum_{i}\sqrt{{\left(\nabla_{x}\;\Delta V\right)}_{\iota }^{2}\;+{\left(\nabla_{y}\;\Delta V\right)}_{\iota }^{2}}$$

Then, the problem to be solved is the constrained optimization problem, as shown in Equation (12).

(12) $$x_{a}=argmin\;{}_{\Delta V}\;\left(\alpha \;{\left\|\nabla \Delta V\right\|}_{2}\right)\;\mathrm{such}\;\mathrm{that}\;{\left\|A\;\Delta V-\Delta m\right\|}^{2}<p$$

## 5. System Design—Measurement Data

**Figure 4:**
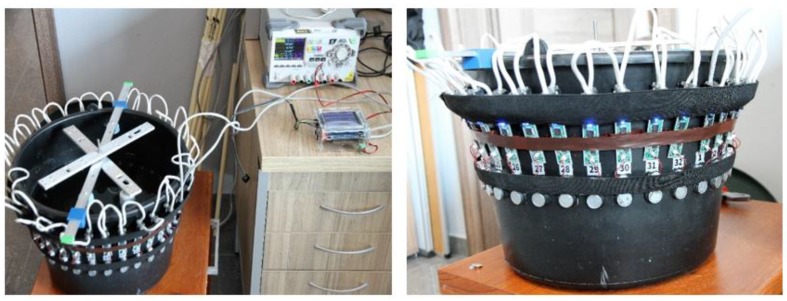
The developed ultrasonic system. A ring of 32 piezoelectric transducers mounted to the black bucket.

The proposed USCT system consists of a big tank of 350 mm with a ring of 32 piezoelectric transducers aligned in a circle, located in a layer of 330 mm inner area diameter, as depicted in Figure 4. These sensors are mounted on the tank, and use a centre frequency of 40 kHz, with a sound pressure level close to 97 dB (30 cm/10 V rms). The reason to choose such a frequency is quite important, relating to the directivity of the emitting sensors. Piezoelectric transducers have a specific pattern of emission, related to their design. While the frequency is going higher, the directivity of sensors becomes narrower. Narrow emission patterns would lead to a sparsely scanned ROI. 

Transducers of MHz of excitation frequency are inappropriate for tomographic application, because of their narrow directivity. Sensors of a few hundred kHz can have better angular coverage. In this work, piezoelectric transducers of 40 kHz are used. The used tank is made from polypropylene profax plastic, due to its low acoustic impedance. The acoustic attenuation of the plastic tank is 5.2 dB/cm with a thickness of 1 cm. The sensors are attached on the outer surface, being non-destructive, pulses travel through the thickness of the tank. It is important for the tank to be composed of “friendly” acoustic materials, so pulses travel without being attenuated.

**Figure 5:**
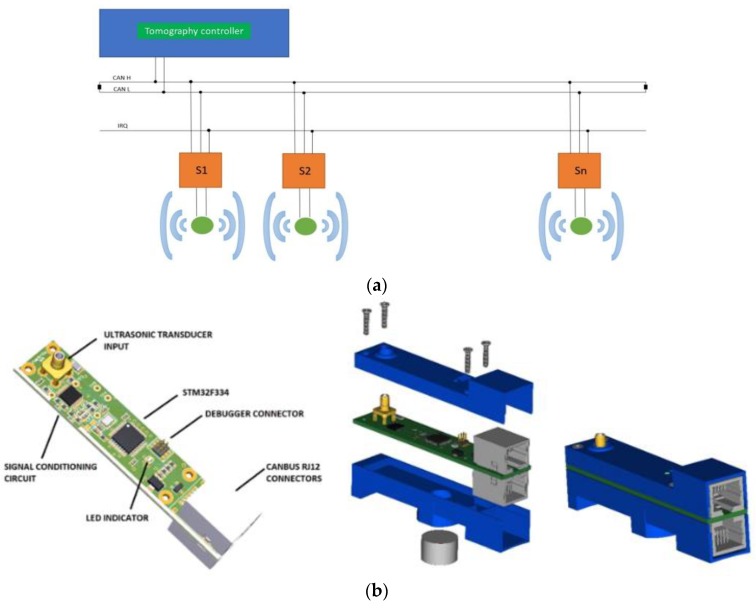
(**a**) Block diagram of the ultrasound tomography. (**b**) Design of active ultrasonic probe.

The concept of the developed ultrasound tomography is based on a parallel data transferring architecture. Its main attribute is the active measurement probes, controlled by, an external module, a CAN bus, as shown in Figure 5a. This is a less time-consuming transferring and receiving data method. This design can exclude a switching part from the system, while the receivers should be in the “receiving mode”. Subsequently, a multiplexer introducing additional delays is neglected. Active measuring probes are divided into digital and analogue parts. The digital part is responsible for sending measurement data to the tomography controller, via the bus. The analogue part has been adapted to work with a piezoelectric transducer operating at 40 kHz, as shown in Figure 5b. The active probe can work both as a receiver of an ultrasonic signal and as a transmitter. The main controller of the tomography is responsible for managing the measurement sequence, setting the active probes in the transmit/receive mode, as well as storing collected results from the other probes. The probes are designed so that they can be placed very close to each other. Power lines, communication buses, and break lines were carried out using RJ-12 cables [34].

**Figure 6:**
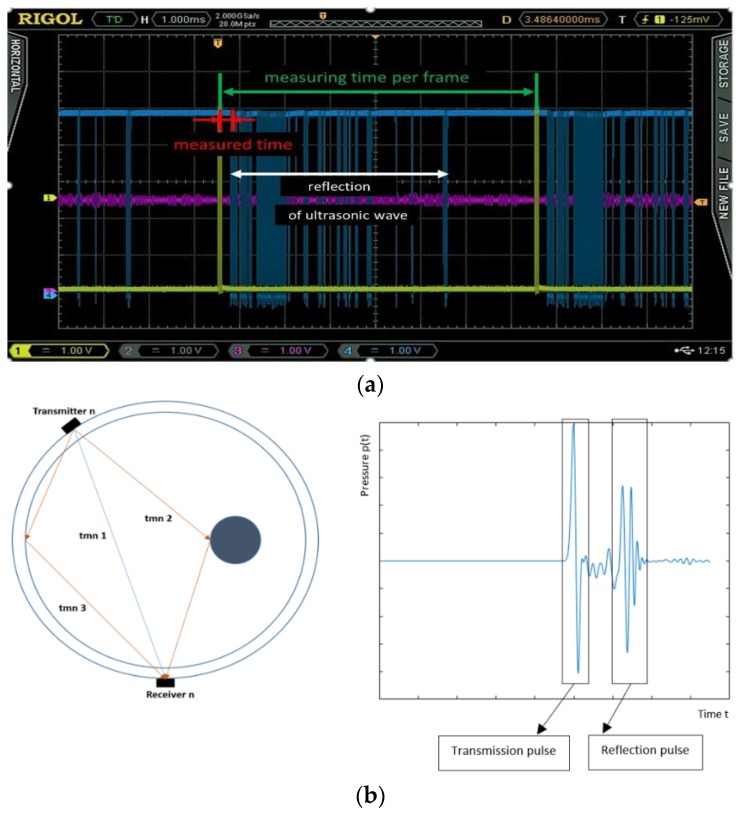
(**a**) Display of transmitted, received, and filtered pulse of a single measuring frame. (**b**) Three possible ways of receiving information by the propagation of the waves. The circular perimeter displays the tank and the dark blue circle inside it displays the object. The form of a received signal.

The time of one measuring frame depends on the ultrasonic reflection and backscattering factor inside the tank. Therefore, consecutive sensor actuations could be undertaken, only once these pulses are completely attenuated. Figure 6a shows the recorded signals and b shows the geometric paths of the transmitted and reflected recorded signals. This is a serious problem, as these reflections extend the time of a single measurement past the time a pulse needs to travel within the tank (transmission time). 

In the case of the test container with 32 measurement probes, obtaining data for one image takes about 240 msec. In this way, the temporal resolution of the system is about 4 frames per second, which also accounts for reconstruction algorithms time to be performed. Ultrasounds as a mechanical wave, while interacting with the material of the domain, could be transmitted, refracted, diffracted, and reflected. All these ultrasounds properties are dependent on the ROI characteristics and materials and type and frequency of the wave. When an incident wave passes through two different materials, its energy transforms to transmitted, refracted, diffracted, and reflected energy according to materials acoustic impedance and the geometry of the event, according to Schnell law. The transmission travel-time tomography is based on the time that the transmitted pulse needs to pass through the scanned area. To compute the sound-speed distribution the system must record the transmitted pulse in each emission frame. When there are big divergences in densities of the ROI, namely, when the materials inside the ROI are in a different phase (liquid/solid), the transmitted mechanical waves lose a lot of their energy due to the great difference in the acoustic impedance of materials under test. Therefore, in such cases, the picked signal is not the transmitted one and subsequently, the sound-speed imaging is not accurate. In conclusion, the proposed sound-speed method can work on cases in which the acoustic impedance does not dramatically change.

### 5.1. TOF Data Acquisition

While an active probe sends an ultrasonic signal of five cycles (tone burst), the rest of the probes are in receiving mode. Active probes measure the time from the moment the signal is sent to when it is picked up by individual transducers (TOF). The sequence repeats until every probe produces a signal, and therefore, their respective times are collected. Figure 6a shows the signal waveform of one pair of transducers. 

A rectangular signal is fed to the ultrasound transducer, in order to force transmission. The obtained signal is also transformed into a rectangular signal (processed signal,) so that it can be read and sent to the microcontroller. The red segment is the measured delay caused by transmission to the control unit. Thanks to the active measuring probes, transfer of analogue signal is reduced to a minimum, and interferences reduced. The probes communicate with the main unit via the digital CAN bus. The concept of an active probe enables the switching system to work independently from the rest of the system.

### 5.2. Filtering Method

USCT devices are each unique, as they intrinsically bear different types or volumes of noise. The “Deleting Outliers” statistical, filtering method was used to handle this noise for all the datasets [34]. D.M. Hawkins describes the notion of an outlier, as an observation that deviates so much from the other observations as to arouse suspicions, considerably, generated by a different mechanism [35]. In our case, “outlier” TOF values usually are generated from back-scattering or reflected signals. An iterative implementation of the Grubbs Test which checks one value at a time, was used to identify the outlier signals. In any given iteration, the tested value is either the highest value, or the lowest, and is the value that is furthest from the sample’s mean [36].

**Figure 7:**
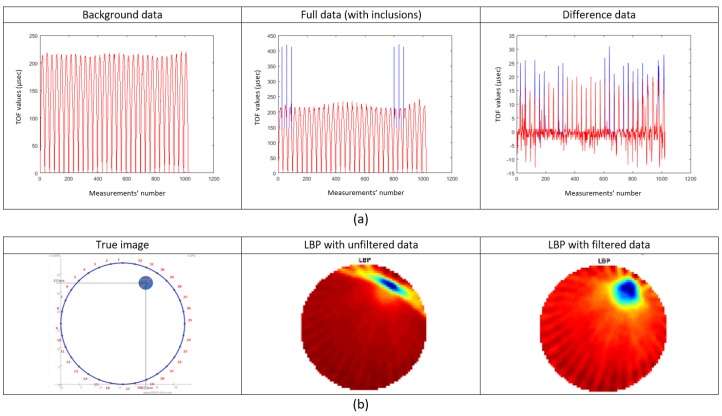
(**a**) Filtering the raw time-of-flight (TOF) data with the statistical method of “deleting outliers” introduced by F. E. Grubbs [35]. (**b**) Reconstruction using a single-step linear back-projection algorithm (LBP) with and without filtering the data.

In Figure 7, the uniform pattern describing the background data, drives us to the conclusion that the developed system provides a high signal-to-noise ratio (SNR). However, unexpected higher values are found in full data. This exposes the system for collection of back-scattered, and reflected pulses. These back-scattered pulses may occur from the surfaces of inclusion(s). This is considered as noise in the transmission tomography data acquisition. In Figure 7a, one can also notice the tremendous effect of the “outlier method”. The blue part of the curves is the part of the curve that has been deleted. This filtering method has been applied to all background, full and, also, difference data before the reconstruction performs. Moreover, reconstructions, using a single-step algorithm such as linear back-projection (LBP), are depicted in Figure 7b. The reconstructions from left to right are using unfiltered data and filtered data, respectively.

## 6. Experimental Results

This section shows the experimental processes and results. Experiments, which are presented in the first section, aim to study the behaviour of the wave propagation in liquid masses using static inclusions of different shapes and setups. The purpose of these experiments is to test the system efficiency in spatial resolution. Factors that affect spatial resolution in USCT are the size and number of sensor elements, according to the dimensions of the ROI, but also to the sensitivity models and regularization algorithms. The experiments that are presented in the second section, are focused on the quantitative information of sound-speed imaging. Our applied experiments are based on the correlation between sound-speed calculated values and liquid density. The “Smoothed Thick Lines” sensitivity matrix method with 90 degrees angle of the beam was used for the reconstructions. An image resolution of (64 × 64) pixels, with pixel spacing to 5.15 mm, was used for visualizing the results. Furthermore, the isotropic TV with a fixed regularization parameter was used for all the experiments, and chosen experimentally.

### 6.1. Qualitative Resolution Experiments

**Figure 8:**
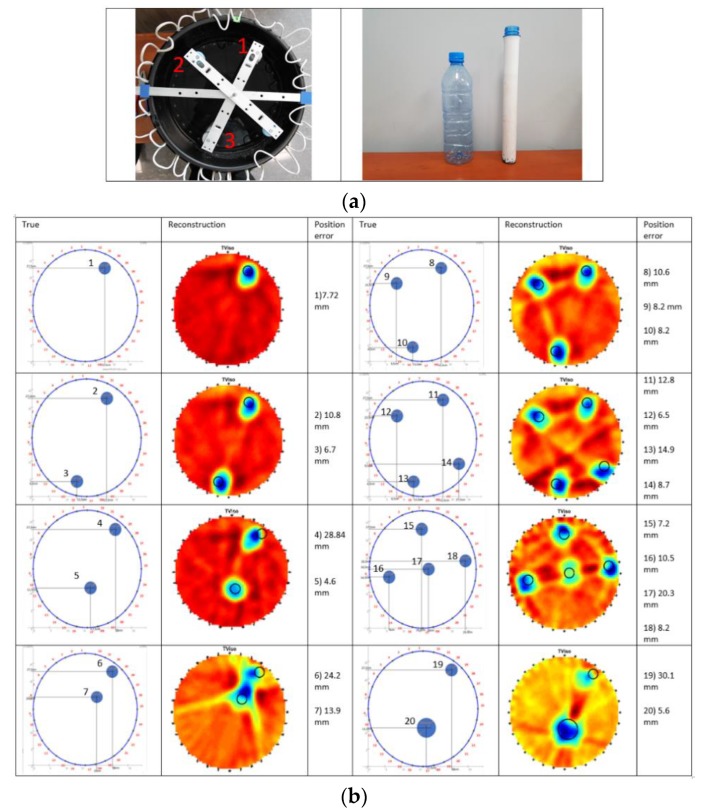
(**a**) Objects used as inclusions in the experiments. (**b**) **Left**: True positions of inclusions. **Middle**: Isotropic total variation reconstruction using a 90 degrees angle beam. **Right**: Position error in mm.

The inclusions consist of cylindrical objects of 2.8 cm (pipe) and 5.8 cm (bottle) diameter. The objects are empty (filled with air). Figure 8a displays the tank with the inclusion mechanisms and the inclusion objects as well. Several disparate positions of the inclusions and some other positions of multiple inclusions are applied to evaluate the reconstruction accuracy of our system, as depicted in Figure 8b. 

Moreover, to thoroughly test the accuracy of algorithms, two objects were placed in different positions, focused on three specific separating distances. The left column presents the geometry of the experiments, the middle column the TV reconstruction, and the right one the position error. Black circles represent the true position of the inclusions, while the red dots represent their centres. Black dots represent the centre of mass of the images. The Euclidian distance between the image centre of mass and the true circle has been calculated. The minimum distance value is 4.6 mm, the maximum is 30.1 mm and the average value of all the distances is 12.4 mm. Air inclusions experiments show a significant low percentage of position error. This is a very good indicator of a system’s accuracy. However, in USCT and especially in travel-time imaging, reflections and back-scattering are always an issue. The more back-scattering effect takes place, the more noise is included in our measurements. Back-scattering is most intense, when objects are placed close to one another. Making reconstruction a more challenging task. 

**Figure 9:**
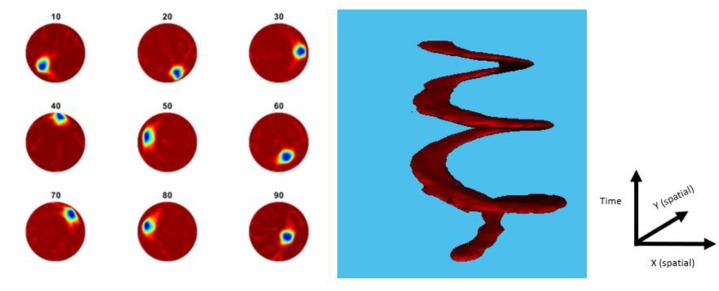
**Left**: Reconstruction of a moving object in 99 locations within the region of imaging in every 10 frames. **Right**: Trajectory of the movement of the sample with shape reconstructed with time (Z axis is time).

Applying many different topologies of multiple inclusions, our method has proved efficient, providing relatively good results. The above experimental results show that our proposed reconstruction methods work well in challenging cases of inclusions, as good quality images could be produced, with a significant low position error. Furthermore, the 32-channel USCT system is tested under a dynamical scenario. Processes occurring in industrial reactors are mostly dynamical, in which stirring is taking place, and therefore, inserting significant disturbances on the pressure field inside the tank. While ultrasound is completely dependent on pressure fields, an important amount of error could arise from measurements. Therefore, an online monitoring system should always overcome this dynamical error factor. To test the response of the system regarding the dynamical error factor, an inclusion was placed in the tank and continuously moved, all while capturing frames. This movement changed the dynamics of the tank and added a significant amount of disturbance to the pressure field. The movement started from the centre to a corner, and then continued around the boundary area in three cycles. Figure 9 displays a dynamic experimental process. A time-variant version of the above TV algorithm was used [37]. The figure shows the reconstruction of a single inclusion moved in 100 locations, within the region of imaging. In this case, data acquisition was performed in situ, while the inclusion was moving. The results are meaningful, in filtering the noise by the moving factor. Moreover, an algorithm of motion tracking was developed, as covered in the latter stage. By post-processing the data captured from the device, the algorithm is able to reconstruct the trajectory of movement of the inclusion over time, as shown in Figure 9. 

### 6.2. Quantitative Resolution Experiments

**Figure 10:**
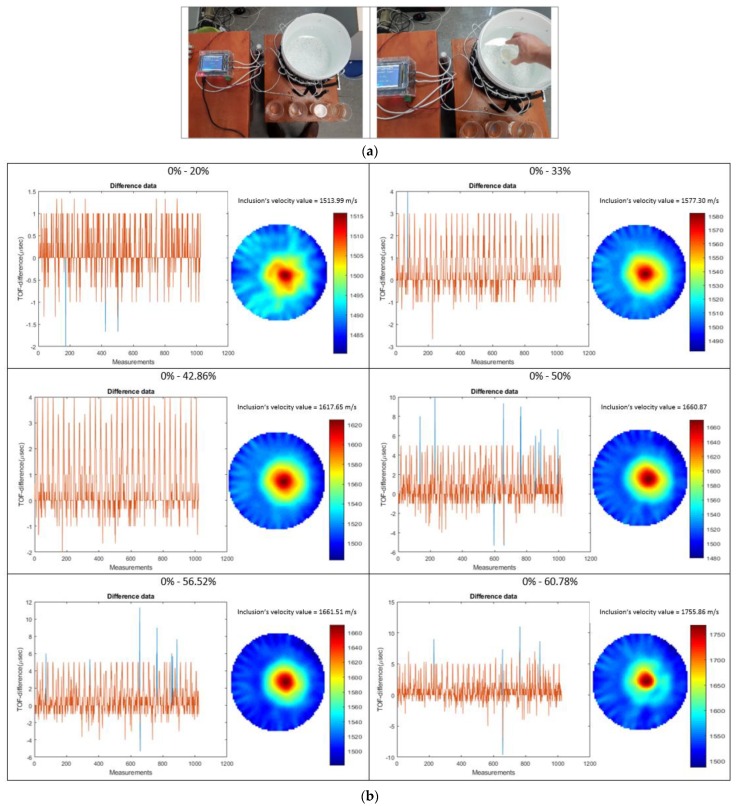
(**a**) Experimental setup. (**b**) Experiment with a slurry mixture of sucrose/water. The scale of reconstructions is in sound-speed units (m/s).

Experiments were carried out to test the response of developed sound-speed imaging algorithms, which could be used in industrial reactors inspecting tool in liquid-mixing processes. This is a preliminary study, based on static experiments of different density liquid characterizations. Density factor is important in many industrial reactors. For example, in a crystallization process, suspension density is increased as crystal yields. Inversely, in a grape fermentation scenario, liquid suspension density is lowered as the sugar turns to alcohol. Liquid density monitoring could positively impact these processes. Moreover, a sound-speed imaging tomographic system would help characterize the uniformity of several binary or ternary mixtures inside industrial tanks. In this way, the stirring process could start as soon as liquid mixture non-uniformity is detected. The stirring in industrial tanks mostly is to make the liquid mixtures be in a uniform state as changes usually occur regionally. Figure 10 depicts the experiments that have been carried out aiming in the sound velocity characterization of liquid solutions with different densities. Sucrose/water solutions were used in different unsaturated concentrations. 

These solutions proved to be a difficult case for characterization because of the small density changes between them. This fact proves the accuracy of the developed TOF sensors and the good quantitative resolution of the tomographic device. Plastic cups filled with a different particle concentration solution each time they were used for this process. The receiving tank was filled with tap water in the room temperature. Background data have been taken using a plastic cup filled with tap water. Full data have been taken using a plastic cup with the sucrose/water binary mixture. Using subtraction imaging, one can achieve to neglect all the cup thin, plastic surface effect in the capturing signals.

Figure 10a displays the experimental process while 10b displays the quantitative imaging results. Six unsaturated solutions of 20%, 33%, 42.86%, 50%, 56.52%, and 60.78% of mass/volume % concentrations of white granulated sugar (sucrose) and tap water at 20 °C were created, while the saturation point of this mixture is 66.7% m/vol. The mass calculated in grams and the volume in mililitres (gr/mL). These solutions were used as inclusions in the tank, which was filled with a medium of tap water at 20 °C. The concentration points were matched with density values, according to the study of Resa et al. [16]. Three consecutive measurement frames have been taken each time and the average value of them is stored. Figure 10b displays, at left, the subtracted TOF data in μs units and at right the sound velocity reconstruction in m/sec units, for every experiment. An adaptive filtering method was applied for the subtracted data. Every value that can be characterized as an outlier and, at the same time, exists fewer times than the ¼ of the total sensor number, is deleted from the dataset. This filtering technique proves to be a consistent and accurate noise removal method. The sound velocity of inclusion calculated using the mean value of the segmented circular area. 

Table 1 provides with the numerical values of the experimental process. The table displays density values related to the binary mixture concentrations, single TOF measurements provided by the tomographic device and calculated sound-speed value provided by the sound-speed imaging software. Density values assigned to different concentrations of sucrose/water binary mixtures based on Resa et al. studies [16]. In this work, Resa et al. calculated a function between density and sucrose concentration in binary unsaturated solutions of sucrose/water. They conducted a single-measurement ultrasonic study on sucrose/water binary mixtures in 30 °C using piezoelectric sensors of 2.5 MHz centre frequency. They evaluated their experimental work by mathematical relations described by Urick’s [17] and Natta-Baccaredda’s [38] prior works. 

These works present formulas that mainly describe the relationship between changes in the chemical composition of solutions and changes in the propagation velocity of sound. Urick’s study of liquid mixtures is based on the linearity of the adiabatic compressibility and density with the volume concentration (13), (14). The sound velocity formula arises in (15):

(13) $$p=\sum_{i}\varphi_{\iota }p_{i}$$

(14) $$k=\sum_{i}\varphi_{\iota }k_{i}$$

(15) $$c=\frac{1}{\sqrt{\left(\sum_{i}\varphi_{i}p_{i}\right)\left(\sum_{i}\varphi_{i}k_{i}\right)}}$$

where c is the sound velocity, k is the adiabatic compressibility, p is the density, and φ is the volume fraction. Based on this study, density values were assigned to the specific concentrations of liquid solutions used in our experimental process. Eventually, the work of Resa et al. is used as a gold standard to evaluate our experimental work. The third column of Table 1 presents the single measurements, which come from the 1st and 16th sensors, accounting for the existence of inclusion in the trajectory of the emitted pulse. The TOF data slightly decreases while the concentration of sucrose becomes higher. The fourth column displays the sound speed scales of all the reconstructions. 

**Figure 11:**
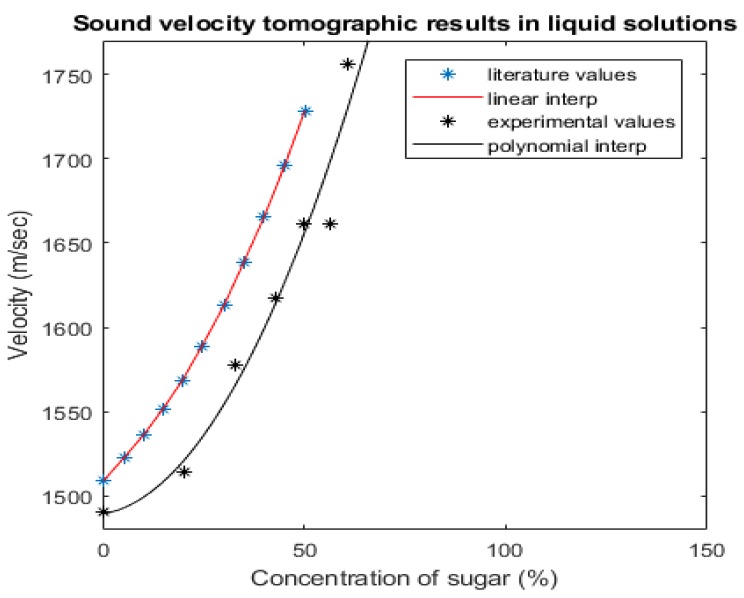
Graph of concentrations values. Experimental values are represented with black dots and single measurement values form literature studies represented with blue dots. Literature values extracted from Resa et al. study [16].

Figure 11 displays with black dots the results related to the experimental work and with blue dots, results extracted by the work of P. Resa et al. [16]. A linear interpolation has been applied to the literature values and a polynomial regression method to the experimental ones. As expected, the results follow an increasing function as the concentration of sucrose increases in the binary mixture. The tomographic graph (black colour) and the “gold standard” graph (red colour) are following the same ascending trend, which is very optimistic. The relatively lower values arise from the fact that the experiments have been occurred in lower temperature close to 20 °C, while the experimental process of Resa et al. occurred at 30 °C. The system provides good absolute values. Translating the concentration values of the sucrose/water solutions to density values, important concepts can be extracted by this work. The developed sound-speed imaging has been proven as having an accurate quantitative resolution. The most challenging reconstruction is the one with the liquid solution of 20% m/vol concentration which leads to a difference in density of 80 kg/m^3^ between the inclusion and the medium. The fact that the developed system can respond so accurately to such challenging changes is very optimistic. Moreover, multiple reconstructions with different concentrations proved a great efficiency of the system to distinguish liquids of density differences up to 40 kg/m^3^, which is assured by the different results between the 50% and 42.86% solutions. 

In lower than 10% concentration change, which means density change of 35 kg/m^3^, such as in the case of 56.52% concentration, the calculated sound velocity value is not perfectly matching the expected one, according to the trajectory of the relative graph. These quantitative results prove great feasibility and efficiency of USCT in characterizing different densities of liquid solutions.

## 7. Conclusions 

This study demonstrated the effectiveness of a newly developed online sound speed travel-time USCT system to inspect liquid mixtures of different densities. The study shows for the first-time sound-speed tomographic imaging to monitor liquid elaboration processes. The system is based on two-dimensional travel-time ultrasound tomography with a central excitation frequency of 40 kHz. Three types of experiments were applied to test the spatial and the quantitative resolution and the system response to a dynamical scenario. The system provides satisfactory results regarding the spatial resolution, being able to detect the position of objects of a few centimetres in size. A dynamical experiment, using a moving object and capturing continuous data, showed the dynamical imaging ability with 4 frames per second speed. Such a temporal resolution is suitable for several industrial processes. Furthermore, the sound-speed imaging provides reliable results for the characterization of liquid solutions based on the distribution of sound velocity distributions. This work provides good performance for sound-speed imaging for solutions with small differences in density. For the first time, meaningful quantitative data have been presented in the industrial USCT field, providing a clear relation between sound-speed imaging and density characterization of liquid solutions. The advantages lie in material distinguishing for liquid mixing cases, in characterizing uniformity of mixtures and finally in calculating approximate sound speed and subsequently density distributions of liquid mixtures. 

## Figures and Tables

**Table 1:** Velocity results from the experimental work.

Numerical Table of Experiments			
Mass Concentration Background—Full Measurements	Density	Single TOF Measurements (1-16 Transducer)	Scale of Reconstruction (Velocity)
0% gr/mL	995.3 (kg/m^3^)	162 μs	-
20% gr/mL	1075 (kg/m^3^)	161 μs	1480–1513.93 m/s
33% gr/mL	1134 (kg/m^3^)	159 μs	1480–1577.31 m/s
42.86% gr/mL	1184 (kg/m^3^)	158 μs	1480–1617.65 m/s
50% gr/mL	1224 (kg/m^3^)	157 μs	1480–1660.87 m/s
56.52% gr/mL	1259 (kg/m^3^)	157 μs	1480–1661.51 m/s
60.78% gr/mL	1284 (kg/m^3^)	156 μs	1480–1755.86 m/s
